# Kinetic Analysis in Horses With Deep Digital Flexor Tendinopathy Within the Digit Diagnosed by Magnetic Resonance Imaging

**DOI:** 10.3389/fvets.2022.893026

**Published:** 2022-05-31

**Authors:** Lori M. Madsen, Santiago D. Gutierrez-Nibeyro, Matthew C. Stewart, Annette M. McCoy, David J. Schaeffer

**Affiliations:** Department of Clinical Veterinary Medicine, College of Veterinary Medicine, University of Illinois Urbana-Champaign, Champaign, IL, United States

**Keywords:** kinetic analysis, force plate, horse, deep digital flexor tendinopathy, magnetic resonance imaging

## Abstract

**Objectives:**

To determine the stance duration and ground reaction forces (GRF) of horses with deep digital flexor (DDF) tendinopathy at the level of the foot and compare the stance duration and GRF to those of clinically sound horses.

**Design:**

Prospective clinical study.

**Animals:**

Sixteen horses (seven horses with bilateral forelimb lameness, four horses with unilateral forelimb lameness, and five horses with no lameness).

**Procedures:**

Analyses of kinetic variables were performed on both forelimbs from sound horses and horses diagnosed with chronic DDF tendinopathy. Stance duration and longitudinal and vertical components of the GRF were determined for the limbs of clinically sound horses and limbs of horses with DDF tendinopathy. Separate Spearman correlation analyses were used to assess potential association within groups (combined left and right forelimbs of clinically sound horses, lamest limbs of horses with DDF tendinopathy, and contralateral limbs of horses with DDF tendinopathy) and with the set of kinetic variables. Analysis of variance on mean ranks of tied values was used to determine differences in kinetic variables between groups (PROC GLIMMIX) using the kinetic values of the clinically sound horses as the reference group.

**Results:**

There were a total of 11 lame horses. Seven horses had bilateral forelimb lameness and four had unilateral lameness. Of the 11 horses, there were 15 DDF tendinopathies. There were eight dorsal border DDF tendinopathies, five core DDF tendinopathies, and two sagittal/parasagittal splits DDF tendinopathies. The most lame limbs of horses with DDF tendinopathy had significantly smaller values for peak vertical force and time of peak braking force than did forelimbs of clinically sound horses. Also, the most lame limbs of horses with DDF tendinopathy had significantly larger values for the time of peak vertical force than did forelimbs of clinically sound horses.

**Conclusions and Clinical Relevance:**

Horses with chronic DDF tendinopathies develop certain alterations of GRF parameters. This information can be used in future studies to determine if particular kinetic variable changes in horses with DDF tendinopathies differ from those of horses with other pathologies within the foot and therefore could be diagnostic.

## Introduction

Deep digital flexor (DDF) tendinopathies are a common injury in horses with forelimb lameness localized to the foot that do not have radiographic abnormalities (1). Diagnosis of DDF tendinopathies within the foot can be challenging because many of these lesions typically cannot be diagnosed with radiography or ultrasonography (1). Magnetic resonance imaging (MRI) is a sensitive diagnostic imaging modality for identifying equine soft tissue pathologies within the foot, including DDF tendinopathies (1); therefore, MRI is typically recommended for horses with lameness localized to the foot and unremarkable or equivocal radiographic findings (2). However, MRI evaluation may not be an option in certain clinical situations due to health or financial reasons.

Locomotion analysis is an important research focus in veterinary medicine, in both experimentally-induced musculoskeletal diseases and in horses with naturally occurring musculoskeletal disease, to determine if this modality can be used clinically to identify the specific location of lesions within the limb (3). Kinetic gait analysis involves measuring the locomotor forces or ground reaction forces (GRF) on the limbs during the stance phase of the stride using a stationary force plate (4). Changes in GRF have been demonstrated using various models of experimentally-induced lameness, including pressure on the hoof sole (5), collagenase-induced superficial digital flexor tendonitis (6) and suspensory desmitis (7), and surgical creation of cartilage defects (8). However, GRF alterations in horses with DDF tendinopathies within the digit have not been investigated.

Kinetic gait analysis relies on biomechanical principles associated with lameness, and is a very sensitive indicator of weight-bearing asymmetries in horses at the walk and trot (5, 6, 9–11). The GRF and stance time in horses with DDF tendinopathies might predict if these horses develop a specific gait or locomotor abnormality that can be further investigated. Charecterization of the altered kinetic variables and stance duration in horses with DDF tendinopathy-mediated lameness within the digit could improve diagnostic evaluation of these cases and response to therapeutic intervention. The purpose of the study was to report the representative GRF of 11 horses with DDF tendinopathies diagnosed by MRI and compare these kinetic variables to those of five clinically sound horses. We suggest this information might be useful clinically, in addition to subjective clinical evaluation, for reliable detection, quantification, and differentiation from other foot pathologies in lame horses. We hypothesized that certain components of the GRF are significantly altered in the lamest limb of horses with DDF tendinopathies.

## Materials and Methods

All procedures were approved by the Institutional Animal Care and use Committee at the University of Illinois Urbana-Champaign and informed consent was obtained from the owners.

### Lame Horses

Inclusion criteria for the study consisted of horses with either a bilateral or unilateral forelimb lameness localized to the foot that were undergoing MRI evaluation. Horses with forelimb lameness were included in the study following physical, lameness, and MRI examinations of both fore feet at the University of Illinois Veterinary Teaching Hospital. Horses had chronic (>6 weeks duration) moderate [grade 2–3 out of 5 in the American Association of Equine Practitioners grading scale (12)] unilateral or bilateral forelimb lameness, had a positive response to lower limb flexion bilaterally, and had no definitive diagnosis based on radiographic evaluation of the lame foot/feet. Lameness evaluation of all lame horses consisted of walking and trotting the horses on hard and soft ground in a straight line and circles in both directions, in addition to jogging the horses over the large animal force plate to obtain GRF. Anesthesia of the palmar digital nerves resolved or markedly improved (90% improvement) the lameness in a straight line and circles in both directions in all lame horses. For horses with bilateral forelimb lameness, the lamest, or more lame limb was resolved or markedly improved first, with anesthesia of the palmar digital nerves, followed by anesthesia of the palmar digital nerves in the contralateral limb.

Of the 11 horses with DDF tendinopathies, nine horses had both forefeet evaluated with a 3.0 T MRI system (Siemens Magnetom Skyra, Siemens Medical Solutions, PA, United States), and two horses had both forefeet evaluated with a 0.25 T MRI system (Vet-MR Grande System, Universal Medical Systems, Inc., OH, United States). A dedicated equine foot coil (0.25 T) or a flexible body coil (3.0 T) were used to obtain the magnetic resonance images. High-field MRI sequences included proton density (PD) fat-saturated Dixon (sagittal plane), T2 Dixon turbo spin-echo (TSE) (transverse plane), short τ inversion recovery (STIR) (all imaging planes), PD TSE (all imaging planes), T1 VIBE (dorsal plane), and T2 3D-gradient echo (all imaging planes). Low-field MRI sequences obtained included T1 3D-gradient echo, T2 TSE, PD, and STIR in all imaging planes. Magnetic resonance images were reviewed by a board-certified large animal radiologist and/or board-certified large animal surgeon. The MRI abnormalities identified were reported using a standardized set of scoring criteria (2).

### Clinically Sound Horses

Horses were healthy, had no history of lameness and were client owned. Horses were determined to be free of lameness by two equine clinicians (grade 0 out of 5 in the American Association of Equine Practitioners grading scale) (12). Evaluations performed to rule out forelimb lameness consisted of walking and trotting the horses on hard and soft ground in a straight line and circles in both directions, in addition to jogging the horses over the large animal force plate to obtain GRF. Young Quarter Horses were chosen as reference for sound horses due to their similar body conformation to those typically affected by unilateral or bilateral forelimb DDF tendinopathy.

### Force Plate Gait Analysis

The testing area for kinetic gait analyses had a 25-m-long runway covered with a rubber mat (Anti-Fatigue Mat, 9RHZ4, Grainger, Palatine, IL) and a 60 × 120-cm force plate (Force plate, EQ6001200-4000, AMTI Inc, Watertown, MA) embedded in the center of the runway. One experienced handler trotted all horses allowing each horse to choose its comfortable speed. Each data collection trial was considered successful when a targeted trotting speed (2.5–3.5 m/s) and acceleration or deceleration (±0.5 m/s^2^) were achieved, and only when the ipsilateral forelimbs and hind limbs fully contacted the force plate; trotting speed (m/s) was measured with infrared sensors (Infrared photoelectric sensor, MEK-92-PAD, Mekontrol Inc, Northborough, MA) connected to the computer analysis system. Ten successful trials (five for each forelimb) per horse were collected and analyzed.

GRF data were sampled at 500 Hz for each successful trial (Acquire, version 7.33, Sharon Software Inc, Owosoo, MI) and calculated with commercially available specialized software (Microsoft Office Excel 2021, Microsoft Corporation, Redmond, WA). The GRF were broken down into vertical and longitudinal (braking or propulsive) components, normalized to the horse's body mass and reported as Newtons per kilogram (N/kg). Impulse values of vertical and longitudinal forces (area under the force to-time curves) were calculated by time integration, then normalized by the horse's body mass and reported as Newton-seconds per kilogram (N·s/kg). In addition, total stance time (time elapsed from initial ground contact to lift-off) was expressed as ms, and the individual' time at which the reaction force peak amplitudes occurred (peak-time position) were expressed in ms. Therefore, the following data were reported: peak braking force (N/kg), braking impulse (N·s/kg), time of peak braking force (ms), peak propulsive force (N/kg), propulsive impulse (N·s/kg), time of peak propulsive force (ms), peak vertical force (N/kg), vertical impulse (N·s/kg), and time of peak vertical force (ms). Only the vertical and longitudinal force components were studied because previous force plate studies have shown that weight-bearing lameness affected mainly those two GRF, whereas changes in the transverse GRF are negligible (13).

The kinetic variable means for each forelimb were the representative data for that horse's limb. For the sound horses, asymmetry indices for the stance time, peak vertical force, and vertical impulse were calculated as described by Weishaupt et al. and expressed as a percentage (14). Using this approach, asymmetric stance time, peak vertical force, or vertical impulse yielded an asymmetry index equal to zero (15). In contrast, a higher peak force (or impulse) in either the left or right forelimb yields a positive or negative asymmetry index (15). These kinetic variables were selected because they are very sensitive for forelimb lameness in horses (16). In horses without lameness and with symmetric interlimb timing, the stance time, peak vertical force, and vertical impulse asymmetry indices are <2.5% (9, 15). Subsequently, data for kinetic variables obtained from five successful trials for each pair of forelimbs were averaged, and the mean values were used to compare the force plate gait analysis sessions.

### Statistical Analysis

Normality of the data for stance time, peak vertical force, and vertical impulse obtained from the clinically sound horses was determined by the Shapiro-Wilk and Anderson-Darling tests. Means of these three kinetic variables were used to calculate the asymmetry indices (ASI) of both forelimbs in the clinically sound horses (15). The same statistical quantities are required for comparing groups, but the kinetic variables from the lame horses were not normally distributed. Hence, all the kinetic variables for the lame and clinically sound horses were summarized and reported as median and range values. Separate Spearman correlation analyses were used to assess potential association within groups (combined left and right forelimbs of clinically sound horses, lamest limbs of horses with DDF tendinopathy, and contralateral limbs of horses with DDF tendinopathy) and with the set of kinetic variables. Analysis of variance on mean ranks tied values was used to determine differences in kinetic variables between groups (PROC GLIMMIX) using the kinetic values of the clinically sound horses as the reference group (17). Analyses used SAS 9.4 (SAS Institute, Cary, NC), and *p* ≤ 0.05 was considered a statistically significant difference.

## Results

All five clinically sound horses included in the study were client owned and were barefoot at the time of the study. The age range of the five horses was 3 to 4 years (median age, 3 years) and body weight range was 445 to 474 kg (median weight, 473 kg); there were three geldings and two mares. All five horses were Quarter Horses. During the study, all sound horses remained free of lameness and had a high degree of symmetry of stance time, peak vertical force, and vertical impulse. In these horses, the forelimb asymmetry indices for the vertical forces ranged from 0.69 to 0.7%.

All horses diagnosed with DDF tendinopathies were lame at the time of the study; seven horses had bilateral forelimb lameness, and four horses had unilateral forelimb lameness. All horses were client owned and barefoot. The age range of the 11 horses was 7 to 12 years (median age, 12 years), and body weight range was 395 to 716 kg (median weight, 527 kg); there were four geldings, one stallion, and six mares. There were six Quarter Horses, two American Paint Horses, one Appaloosa, one Arabian, and one Saddlebred. The MRI abnormalities identified in the forelimbs of these horses are summarized in Table 1. There were eight dorsal border DDF tendinopathies, five core DDF tendinopathies, and two sagittal/parasagittal split DDF tendinopathies. Dorsal border DDF tendinopathies were identified at the level of the collateral sesamoidean ligament in three horses, the proximal recess of the navicular bursa in two horses, the navicular bone in two horses, and in one horse extended from the proximal phalanx to the level of the proximal recess of the navicular bursa. Core DDF tendinopathies were identified at the level of the proximal interphalangeal joint in two horses, and at the insertion of the DDF tendon in one horse. In one horse, the lesion extended from the collateral sesamoidean ligament to the navicular bone and, in another case, extended from the middle phalanx to the level of the navicular bone. Sagittal/parasagittal split DDF tendinopathies were identified between the navicular bone and the insertion of the DDF tendon in one horse and, in a second horse, extended from the collateral sesamoidean ligament to the insertion of the DDF tendon. There were always concurrent pathologies reported in the lamest limb of horses with DDF tendinopathies. These included navicular bone abnormalities (one horse), navicular bone and navicular bursa abnormalities (three horses), and navicular bone, navicular bursa, and distal sesamoidean impar ligament abnormalities (seven horses).

**Table 1:** Magnetic resonance imaging abnormalities identified in the 11 lame horses with deep digital flexor tendinopathies.

**Abnormality identified**	**Lamest** **limb**	**Contralateral limb**
Altered signal intensity within the trabecular bone of the NB	10	5
Enlarged synovial invaginations in the distal border of the NB	4	2
NB flexor surface erosion	5	0
Effusion and synovitis of the distal interphalangeal joint	2	2
Core DDF tendinopathy	3	2
Sagittal/parasagittal DDF tendinopathy	2	0
Dorsal border DDF tendinopathy	6	2
Navicular bursa effusion and synovitis	2	2
Navicular bursa synovial proliferation and synovial membrane thickening	8	3
Collateral enthesopathy of the distal interphalangeal joint	1	1
Distal sesamoidean impar desmopathy	2	1
Distal sesamoidean impar enthesopathy	4	1
Collateral sesamoidean desmopathy	3	1

*DDF, deep digital flexor; NB, navicular bone*.

*The numbers in the columns represent the total number of abnormalities identified*.

There was no significant difference in the median trotting speed (*p* = 0.58) between sound and lame horses (2.80 ± 0.24 and 2.95 ± 0.29 m/s, respectively). There was also no significant difference (*p* = 0.87) in the median stance duration between lamest limb of horses with DDF tendinopathy and forelimbs of sound horses (Table 2). The lamest limbs of horses with DDF tendinopathy had significantly lower values for peak vertical force and time of peak braking force than did the forelimbs of clinically sound horses. Also, the lamest limbs of horses with DDF tendinopathy had significantly higher values for the time of peak vertical force than did forelimbs of clinically sound horses.

**Table 2:** Median and range forelimb kinetic gait variables obtained from 11 lame horses and 5 healthy sound horses during force plate trials (10 trials/horse) performed with the horses barefoot.

**Variable**	**Sound horses**	**Lame horses**	
	**(forelimbs combined)**	**lamest limb**	**contralateral limb**
Stance time (ms)	320 (262–368)	344 (287–384)	328 (270–397)
Peak braking force (N/Kg)	−0.58 (0.17–0.98)^a^	−0.74 (0.13–1.2)^b^	−1.2 (0.16–1.4)^c^
Braking impulse (N·s/kg)	−0.05 (0.01–0.12)	−0.08 (0–0.13)	−0.1 (0–0.16)
Time of peak braking force (ms)	124 (76–175)^a^	43 (12–151)^b^	89 (13–169)
Peak propulsive force (N/Kg)	0.45 (0.21–0.92)^a^	0.84 (0.19–1.15)^b^	0.82 (0.17–1.24)
Propulsive impulse (N·s/kg)	0.03 (0.01–0.13)^a^	0.07 (0.02–0.12)^b^	0.06 (0.01–0.11)
Time of peak propulsive force (ms)	227 (161–277)	253 (180–375)	232 (195–272)
Peak vertical force (N/Kg)	9.69 (8.81–11.16)^a^	9.09 (6.08–10.79)^b^	10.65 (8.41–11.46)^c^
Vertical impulse (N·s/kg)	1.93 (1.72–2.08)	1.90 (1.32–2.11)	2.05 (1.64–2.34)
Time of peak vertical force (ms)	157 (138–233)^a^	165 (133–209)^b^	153 (130–223)

*Kinetic gait analysis was performed at a trot for 5 valid repetitions, and kinetic parameters were calculated. N/kg=Newtons per kilogram; N·s/kg= Newton-seconds per kilogram. Ms = ms*.

a, b, c *Within a row, values with different superscript letters are significantly (p <0.05) different*.

## Discussion

Although the lame horses had multiple MRI abnormalities diagnosed in the lamest (or lame) limb, the most clinically significant abnormalities in all 11 horses were DDF tendinopathies. Not surprisingly, the peak vertical force was significantly lower in the lamest limb than in the contralateral limb or in sound horses. Generally, with increasing lameness, horses exhibit lower peak vertical forces of the painful limb to reduce structural stress (15). Also, the median vertical impulse was not significantly different in the lamest limb compared to the contralateral limb, and to sound horses. This finding is consistent with previous publications that have demonstrated that the vertical impulse is maintained in lame limbs while reducing the peak vertical force, by increasing stance duration (15).

The time of peak vertical force, was significantly increased in the lamest limb compared to the contralateral limb and to sound horses, indicating a reduced rate of loading. This is consistent with the findings of Weishaupt et al. who reported that horses reduce the rate of loading and prolong their stance time as a mechanism to reduce the vertical peak force of the lame limb (15). The stance time was increased in the lamest and contralateral limb of horses with DDF tendinopathy compared to the the clinically sound horses. This is consistent with previous studies that reported a prolonged stance time reduces peak vertical forces on the lame limb (15). However, in the current study the increase in stance time did not reach statistical significance. It is possible that statistical significance was not achieved due to the low number of horses included in the study.

The time of peak braking force was significantly less in the lamest limb of horses with DDF tendinopathies than the contralateral limb and in sound horses indicating an increased rate of deceleration. Several horses in the study had erosions on the flexor surface of the navicular bone and likely had adhesions between the DDF tendon and navicular bone. We suggest the adhesions could have decreased the elasticity of the DDF tendon and produced a faster time of braking force peak.

Braking impulse and peak braking force were not statistically significant among the lamest limb, contralateral limb, or sound horses. This is in contrast to previously reported studies that found braking impulse and peak braking forces were significantly decreased in the lame limb and increased in the contralateral limb (6, 8). The dissimilarities in braking impulse and peak braking force between the current and previously reported studies (6, 8). may be due to differences in the sources of lameness or the populations of horses. Morris et al. reported on a model of experimentally induced lameness with the surgical creation of a cartilage defect and Clayton et al. reported on experimentally induced lameness with a collagenase-induced superficial digital flexor tendonitis model. The population of horses in the current study evaluated naturally occurring chronic lameness predominantly involving the DDF tendon. In addition, the population of horses used by Morris et al. consisted primarily of Standardbreds and the population of horses used by Clayton et al. were Warmbloods, where as our study consisted of several breeds with Quarter Horses predominating.

There were several limitations of this study. First, the low number of lame horses recruited into the study may have resulted in type-2 statistical error. Second, horses with DDF tendinopathies had other abnormalities identified on MRI which could have contributed to the changes in gait to some degree. Third, the lame horses were a combination of unilateral and bilateral lameness which may have altered kinetic variables which we are unable to identify. Fourth, the clinically sound horses did not have MRI evaluation of the fore feet to rule out DDF tendinopathies. Fifth, there were fewer control horses than lame horses and they were not age matched. Sixth, we did not have a group of lame horses with other soft tissue abnormalities (i.e., collateral desmopathy of the distal interphalangeal joint) and without DDF tendinopathies to determine if alteration of certain components of the kinetic variables reported in our study were strictly related to the DDF tendinopathies. Eighth, hoof balance was measured subjectively.

In conclusion, horses with chronic DDF tendinopathies develop alteration of certain components of kinetic variables. Future studies are necessary to verify our results and compare the kinetic variables to those of horses with other soft tissue injuries of the foot.

## Data Availability Statement

The raw data supporting the conclusions of this article will be made available by the authors, without undue reservation.

## Ethics Statement

The animal study was reviewed and approved by the Institutional Animal Care and Use Committee at the University of Illinois Urbana-Champaign. Written informed consent was obtained from the owners for the participation of their animals in this study.

## Author Contributions

LM and SG-N contributed to study design, data collection, data interpretation, and manuscript preparation. MS and AM contributed to data interpretation and manuscript preparation. DS contributed to data analysis and interpretation, and manuscript preparation. All authors approved the final manuscript.

## Conflict of Interest

The authors declare that the research was conducted in the absence of any commercial or financial relationships that could be construed as a potential conflict of interest.

## Publisher's Note

All claims expressed in this article are solely those of the authors and do not necessarily represent those of their affiliated organizations, or those of the publisher, the editors and the reviewers. Any product that may be evaluated in this article, or claim that may be made by its manufacturer, is not guaranteed or endorsed by the publisher.
